# Supplementary material for: Isolation and Genome-Based Characterization of Biocontrol Potential of Bacillus siamensis YB-1631 against Wheat Crown Rot Caused by Fusarium pseudograminearum
Source: J Fungi (Basel). 2023 May 9;9(5):547. doi: 10.3390/jof9050547 (PMC10219336; doi:10.3390/jof9050547)
Supplement: Supplementary file 1 [file jof-09-00547-s001.zip › jof-2232543-supplementary.pdf]

Table S1. Characteristics of the *Bacillus siamensis* YB-1631 genome

Genome size (bp)	Gene number	Coding sequence length (bp)	Average gene length (bp)	GC% (coding region)	Coding sequence length as % of total genome
4090312	4357	3629469	833.02	46.72	88.73

Table S2. RNA genes in the *Bacillus siamensis* YB-1631 genome

Group	Type	Number	Total length (bp)	Average Length (bp)	%of genome
rRNA	5s rRNA	9	1035	115	0.03
	16s rRNA	9	13842	1538	0.34
	23s rRNA	9	26343	2927	0.64
tRNA	tRNA	87	6710	77.13	0.16
potential RNA	antisense	2	196	98	0
	leader	23	3,905	169.78	0.1
	riboswitch	32	3,805	118.91	0.09
	ribozyme	1	382	382	0.01
	sRNA	16	2,167	135.44	0.05
	thermoregulator	1	365	365	0.01
	other	14	2,129	152.07	0.05

Table S3. Gene islands, CRISPR and prophage structures in the *Bacillus siamensis* YB-1631 genome

Type	ID	Start	End
Gene Islands	Island1	509,762	547,584
	Island2	909525	926795
	Island3	937829	951065
	Island4	1394409	1401748
	Island5	2095249	2241222
	Island6	2734836	2772948
	Island7	2807447	2815838
	Island8	3181581	3218950
	Island9	3256082	3267849
	Island10	3280447	3317632
CRISPR	crispr1	478211	478485
	crispr2	748608	748768
	crispr3	795967	802434
	crispr4	1292613	1293475
	crispr5	2947347	2947609
	crispr6	3659250	3659460
Prophage Structures	prophage1	610,700	651,704
	prophage2	904,145	924,584
	prophage3	1,278,292	1,304,778
	prophage4	2,100,862	2,138,318
	prophage5	2,149,864	2,173,358
	prophage6	2,207,250	2,229,404
	prophage7	2,732,480	2,756,485
	prophage8	3,185,174	3,205,095
	prophage9	3,282,530	3,302,646

Table S4. Functional annotation of the *Bacillus siamensis* YB-1631 genome

Database	Annotated number	Unannotated number
**NR**	4194(96.26%)	163(3.74%)
**SwissProt**	3608(82.81%)	749(17.19%)
**COG**	3174(72.85%)	1183(27.15%)
**KEGG**	2229(51.16%)	2128(48.84%)
**GO**	3114(71.47%)	1243(28.53%)
**Total**	4197(96.33%)	160(3.67%)

Table S5 Genes for antimicrobial compounds in the *Bacillus siamensis* YB-1631 genome

Function	Gene in strain YB-1631	Gene name	Protein Accession Number	Description	Identity	E value
Cluster 1: Nonribosomal peptide synthesis (surfactin)	orf00328	yciC	P94400|YCIC_BACSU	Putative metal chaperone YciC	327/398(82.16)	4.83E-240
	orf00571	yx01	O06012|ADHB_BACSU	Uncharacterized zinc-type alcohol dehydrogenase-like protein AdhB	352/378(93.12)	3.71E-268
	orf00330	yckC	P42401|YCKC_BACSU	Uncharacterized protein YckC	96/151(63.58)	7.12E-67
	orf00331	yckD	P42402|YCKD_BACSU	Uncharacterized protein YckD	64/110(58.18)	5.72E-34
	orf00348	yczE	O34927|YCZE_BACSU	Uncharacterized membrane protein YczE	151/202(74.75)	4.25E-107
	orf00333	nin	P12669|NIN_BACSU	DNA-entry nuclease inhibitor	98/132(74.24)	1.75E-69
	orf00334	nucA	P12667|NUCA_BACSU	DNA-entry nuclease	100/141(70.92)	2.49E-71
	orf00336	hxlB	P42404|PHI_BACSU	3-hexulose-6-phosphate isomerase	135/185(72.97)	4.18E-91
	orf00337	hxlA	P42405|HPS_BACSU	3-hexulose-6-phosphate synthase	184/209(88.04)	1.23E-123
	orf00338	hxlR	P42406|HXLR_BACSU	HTH-type transcriptional activator HxlR	102/119(85.71)	1.12E-73
	orf00341	srfAA	P27206|SRFAA_BACSU	Surfactin synthase subunit 1	2720/3591(75.74)	0
	orf00342	srfAB	Q04747|SRFAB_BACSU	Surfactin synthase subunit 2	2766/3587(77.11)	0
	370593..370733	comS	P80355	Competence protein S	46/46(100)	0
	orf00343	srfAC	Q08787|SRFAC_BACSU	Surfactin synthase subunit 3	1117/1270(87.95)	0
	orf00344	srfAD	Q08788|SRFAD_BACSU	Surfactin synthase thioesterase subunit	187/236(79.24)	2.50E-141
	orf00346	aspC	Q55128|AAT_SYNY3	Aspartate aminotransferase	66/256(25.78)	7.99E-12
	orf00347	sfp	P39144|LP14_BACIU	4'-phosphopantetheinyl transferase	214/223(95.96)	3.95E-163
	orf00348	yczE	O34927|YCZE_BACSU	Uncharacterized membrane protein YczE	151/202(74.75)	4.25E-107
	orf00349	tcyC	P39456|TCYC_BACSU	L-cystine import ATP-binding protein TcyC	233/247(94.33)	1.70E-164
	orf00350	tcyB	P42200|TCYB_BACSU	L-cystine transport system permease protein TcyB	209/229(91.27)	2.26E-143
Cluster 2: Nonribosomal peptide synthesis (fengycin)	orf02024	dacC	P39844|DACC_BACSU	D-alanyl-D-alanine carboxypeptidase DacC	295/491(60.08)	2.24E-219
	orf02022	fenC	WP_013352476.1	non-ribosomal peptide synthase FenC	1542/2602(59.3)	2.24E-219
	orf02022	fenA	WP_269774982.1	non-ribosomal peptide synthase FenA	1784/2581(69.1)	2.50E-141
	orf02022	fenD	WP_269774988.1	non-ribosomal peptide synthase FenD	1918/2578(74.4)	6.86E-199
	orf02021	fenE	WP_049628582.1	non-ribosomal peptide synthase FenE	1200/1269(94.60)	0
	orf02020	yngL	O34506|YNGL_BACSU	UPF0713 protein YngL	91/130(70.00)	4.01E-57
	orf02018	yngK	O35015|YNGK_BACSU	Glycosyl hydrolase YngK	378/514(73.54)	4.54E-281
	orf02017	yngJ	O34421|ACDC_BACSU	Probable acyl-CoA dehydrogenase YngJ	334/380(87.89)	8.69E-236
	orf02016	yngI	O31826|YNGI_BACSU	Putative acyl-CoA synthetase YngI	398/546(72.89)	8.02E-305
	orf02015	yngH	O34544|ACCC2_BACSU	Biotin carboxylase 2	328/441(74.38)	1.45E-235
	orf02014	ND	C0H419|BLAP_BACSU	Biotin/lipoyl attachment protein	51/66(77.27)	4.10E-31
	orf02013	yngG	O34873|HMGCL_BACSU	Hydroxymethylglutaryl-CoA lyase YngG	205/298(68.79)	7.27E-148
	orf02012	yngF	O34893|YNGF_BACSU	Putative enoyl-CoA hydratase/isomerase YngF	178/259(68.73)	5.39E-114
	orf02011	yngE	O31825|YNGE_BACSU	Uncharacterized carboxylase YngE	431/509(84.68)	0
Cluster 3:	orf03339	*ald*	Q08352|DHA_BACSU	Alanine dehydrogenase	337/376(89.63)	6.89E-236

Nonribosomal peptide synthesis（Bacillibactin）	orf03340	yukJ	C0SPB1丨YUKJ_BACSU	Uncharacterized protein YukJ	178/225(79.11)	4.98E-137
	orf03341	mbtH	C0H3Q6丨YBDZ_BACSU	Uncharacterized protein YbdZ	38/67(56.72)	2.80E-24
	orf03342	dhbF	P45745丨DHBF_BACSU	Dimodular nonribosomal peptide synthase	1657/2378(69.68)	0
	orf03343	dhbB	P45743丨DHBB_BACSU	Isochorismatase	227/314(72.29)	3.84E-167
	orf03344	dhbE	P40871丨DHBE_BACSU	2,3-dihydroxybenzoate-AMP ligase	435/533(81.61)	0
	orf03345	dhbC	P45744丨DHBC_BACSU	Isochorismate synthase DhbC	260/395(65.82)	1.03E-187
	orf03346	dhbA	P39071丨DHBA_BACSU	2,3-dihydro-2,3-dihydroxybenzoate dehydrogenase	186/261(71.26)	4.31E-128
	orf03347	besA	O32102丨BESA_BACSU	Ferri-bacillibactin esterase BesA	174/269(64.68)	7.88E-126
	orf03348	yuiH	O32103丨YUIH_BACSU	Uncharacterized oxidoreductase YuiH	170/198(85.86)	5.46E-139
	orf03349	bioYB	O32104丨BIOYB_BACSU	Putative biotin transporter BioYB	145/195(74.36)	3.18E-97
	orf03350	yuiF	O32105丨YUIF_BACSU	Putative amino acid transporter YuiF	398/442(90.05)	2.39E-255
	orf03351	pepA	A7Z8B5丨AMPA_BACVZ	Probable cytosol aminopeptidase	485/496(97.78)	0
Cluster 4: transAT polyketide synthesis (Bacillaene)	orf01886	baeS	O31785丨PKSS_BACSU	Polyketide biosynthesis cytochrome P450 PksS	308/403(76.43)	4.50E-227
	orf01885	baeR	O31784丨PKSR_BACSU	Polyketide synthase PksR	1491/2569(58.04)	0
	orf01884	baeN	O31782丨PKSN_BACSU	Polyketide synthase PksN	3482/5492(63.40)	0
	orf01883	baeM	P40872丨PKSM_BACSU	Polyketide synthase PksM	2171/3519(61.69)	0
	orf01882	baeL	Q05470丨PKSL_BACSU	Polyketide synthase PksL	2858/4558(62.70)	0
	orf01881	baeJ	P40806丨PKSJ_BACSU	Polyketide synthase PksJ	3156/5042(62.59)	0
	orf01880	baeI	P40802丨PKSI_BACSU	Putative polyketide biosynthesis enoyl-CoA isomerase PksI	186/248(75.00)	2.90E-134
	orf01879	baeH	P40805丨PKSH_BACSU	Probable polyketide biosynthesis enoyl-CoA hydratase PksH	169/256(66.02)	1.26E-123
	orf01878	baeG	P40830丨PKSG_BACSU	Polyketide biosynthesis 3-hydroxy-3-methylglutaryl-ACP synthase PksG	350/419(83.53)	1.25E-262
	orf01877	acpK	Q7PC63丨ACPK_BACSU	Polyketide biosynthesis acyl-carrier-protein AcpK	57/81(70.37)	5.42E-34
	orf01876	baeE	A7Z4Y0丨BAEE_BACVZ	Polyketide biosynthesis protein BaeE	700/746(93.83)	0
	orf01875	baeD	A7Z4X9丨BAED_BACVZ	Polyketide biosynthesis acyltransferase homolog BaeD	290/324(89.51)	3.23E-212
	orf01874	baeC	A7Z4X8丨BAEC_BACVZ	Polyketide biosynthesis malonyl CoA-acyl carrier protein transacylase BaeC	207/220(94.09)	1.74E-146
	orf02687	baeB	P54501丨YQGX_BACSU	Probable metallo-hydrolase YqgX	174/212(82.08)	8.46E-132
Cluster 5: transAT polyketide synthesis (difficidin)	orf02580	dfnA	O34787丨PKSE_BACSU	Polyketide biosynthesis protein PksE	453/757(59.84)	0
	orf02579	dfnY	WP_045927167.1	D-fructose-6-phosphate amidotransferase [Bacillus siamensis]	326/326(100.00)	2.77E-240
	orf02578	dfnX	WP_096378593.1	acyl carrier protein [Lysobacter enzymogenes]	25/80(31.25)	2.49E-09
	orf02577	dfnB	P23971丨MENE_BACSU	2-succinylbenzoate--CoA ligase	133/499(26.65)	2.73E-30
	orf02576	dfnC	Q9X248丨FABG_THEMA	3-oxoacyl-[acyl-carrier-protein] reductase FabG	96/240(40.00)	1.38E-57
	orf02575	dfnD	P40872丨PKSM_BACSU	Polyketide synthase PksM	1626/4264(38.13)	0
	orf02574	dfnE	P40806丨PKSJ_BACSU	Polyketide synthase PksJ	892/1874(47.60)	0
	orf02573	dfnF	P40872丨PKSM_BACSU	Polyketide synthase PksM	903/1927(46.86)	0
	orf02572	dfnG	O31782丨PKSN_BACSU	Polyketide synthase PksN	1436/3804(37.75)	0

	orf02571	dfnH	P40806丨PKSJ_BACSU	Polyketide synthase PksJ	833/2413(34.52)	0
	orf02570	dfnI	P40872丨PKSM_BACSU	Polyketide synthase PksM	747/2099(35.59)	0
	orf02569	dfnJ	O31784丨PKSR_BACSU	Polyketide synthase PksR	708/2755(25.70)	3.23E-231
	orf02568	dfnK	O34374丨YJIB_BACSU	Putative cytochrome P450 YjiB	137/355(38.59)	4.21E-86
	orf02567	dfnL	P40830丨PKSG_BACSU	Polyketide biosynthesis 3-hydroxy-3-methylglutaryl-ACP synthase PksG	300/417(71.94)	7.78E-226
	orf02566	dfnM	P40802丨PKSI_BACSU	Putative polyketide biosynthesis enoyl-CoA isomerase PksI	138/245(56.33)	3.94E-94
Cluster 6: polyketide-like synthesis (butirosin A)	orf01106	btrW	O07550丨YHEI_BACSU	Probable multidrug resistance ABC transporter ATP-binding/permease protein YheI	471/585(80.51)	0
	orf01107	btrX	O07549丨YHEH_BACSU	Probable multidrug resistance ABC transporter ATP-binding/permease protein YheH	527/671(78.54)	0
Clusters 7 terpene synthesis (squalene/phytoene)	orf01214	asnO	O05272丨ASNO_BACSU	asparagine synthase (glutamine-hydrolyzing)	523/613(85.32)	0
	orf01215	yisP	O06728丨YISP_BACSU	squalene/phytoene synthase family protein	113/239(47.28)	5.92E-62
	orf01216	yisQ	O07940丨YISQ_BACSU	putative transporter YisQ	403/455(88.57)	1.19E-278
	orf01217	yisR	P40331丨YISR_BACSU	AraC family transcriptional regulator	229/280(81.79)	2.76E-167
Clusters 8 terpene synthesis (squalene-hopene)	orf02090	dhaS	O34660丨ALDH4_BACSU	aldehyde dehydrogenase family protein	452/495(91.31)	0
	orf02092	sqhC	Q796C3丨SQHC_BACSU	squalene--hopene cyclase	218/304(71.71)	4.08E-143
	orf02096	yflN	O34409丨YFLN_BACSU	MBL fold metallo-hydrolase	63/234(26.92)	5.61E-14
Cluster 9 : nonribosomal peptide + polyketide synthesis (bacillomycin D）	orf02001	yxjC	P42314丨YXJC_BACSU	Uncharacterized transporter YxjC	381/480(79.38)	9.51E-254
	orf02000	scoA	P42315丨SCOA_BACSU	Probable succinyl-CoA:3-ketoacid coenzyme A transferase subunit A	192/230(83.48)	6.88E-140
	orf01999	scoB	P42316丨SCOB_BACSU	Probable succinyl-CoA:3-ketoacid coenzyme A transferase subunit B	178/213(83.57)	6.00E-125
	orf01998	yxjF	P42317丨YXJF_BACSU	Uncharacterized oxidoreductase YxjF	192/257(74.71)	4.34E-134
	orf01757	bmyD	P71019丨FABD_BACSU	Malonyl CoA-acyl carrier protein transacylase	286/317(90.22)	6.86E-199
	orf01996	bmyA	Q9R9J1丨MYCA_BACIU	Mycosubtilin synthase subunit A	3158/3984(79.27)	0
	orf01995	bmyB	Q9R9J0丨MYCB_BACIU	Mycosubtilin synthase subunit B	4230/5371(78.76)	0
	orf01994	bmyC	Q9R9I9丨MYCC_BACIU	Mycosubtilin synthase subunit C	1665/2573(64.71)	0
	orf01992	xynD	Q45071丨XYND_BACSU	Arabinoxylan arabinofuranohydrolase	473/513(92.20)	0
	orf01991	ynfF	Q6YK37丨XYNC_BACIU	Glucuronoxylanase XynC	397/423(93.85)	1.70E-305
Cluster 10: nonribosomal peptide + polyketide synthesilocillomycin	orf00189	ybbR	O34659丨CDAR_BACSU	CdaA regulatory protein CdaR	361/418(86.36)	4.61E-254
	orf00190	glmM	A7Z0V3丨GLMM_BACVZ	Phosphoglucosamine mutase	445/448(99.33)	0
	orf00193	glmS	E0U070丨GLMS_BACPZ	Glutamine--fructose-6-phosphate aminotransferase [isomerizing]	559/600(93.17)	0
	orf00195	potA	P42332丨BCRA_BACLI	Bacitracin transport ATP-binding protein BcrA	80/254(31.50)	1.54E-33
	orf00199	kdgR1	P15339丨DKGB_CORSS	2,5-diketo-D-gluconic acid reductase B	143/276(51.81)	4.64E-93

Table S6 Genes for extracellular hydrolases in the *Bacillus siamensis* YB-1631 genome

Function	Gene in strain YB-1631	Gene name	Description	UniProt Accession Number	Identity	E_value
Peptidase	orf00180	amiE	O05213丨AMIE_BACSU	N-acetylmuramyl-L-alanine amidase	322/404(79.70)	5.51E-243
	orf02859	yrvJ	O32041丨YRVJ_BACSU	Putative N-acetylmuramoyl-L-alanine amidase YrvJ	349/522(66.86)	6.12E-243
	orf02620	yqiI	P54525丨YQII_BACSU	Uncharacterized protein YqiI	146/203(71.92)	2.09E-90
	orf03846	lytD	P39848丨LYTD_BACSU	Beta-N-acetylglucosaminidase	259/334(77.54)	2.57E-178
Amidase	orf01073	lytF	O07532丨LYTF_BACSU	Peptidoglycan endopeptidase LytF	370/493(75.05)	8.33E-213
	orf01078	lytE	P54421丨LYTE_BACSU	Probable peptidoglycan endopeptidase LytE	205/334(61.38)	8.94E-127
	orf02024	dacC	P39844丨DACC_BACSU	D-alanyl-D-alanine carboxypeptidase DacC	295/491(60.08)	2.24E-219
	orf02102	cwlS	O31852丨CWLS_BACSU	D-gamma-glutamyl-meso-diaminopimelic acid endopeptidase CwlS	280/419(66.83)	6.91E-189
	orf03752	cwlO	P40767丨CWLO_BACSU	Peptidoglycan DL-endopeptidase CwlO	382/473(80.76)	3.86E-146
	orf03858	pgdS	P96740丨PGDS_BACSU	Gamma-DL-glutamyl hydrolase	274/410(66.83)	3.02E-208
Glycosidase and glycosyltransferase	orf00179	nagZ	P40406丨NAGZ_BACSU	Beta-hexosaminidase	482/638(75.55)	0
	orf02076	yocH	O34669丨YOCH_BACSU	Cell wall-binding protein YocH	217/292(74.32)	5.46E-123
	orf01281	CwlQ	O31608丨YJBJ_BACSU	Bifunctional muramidase/lytic transglycosylase CwlQ	113/134(84.33)	1.75E-74
Protease	orf00011	dacA	P08750丨DACA_BACSU	D-alanyl-D-alanine carboxypeptidase DacA	356/445(80.00)	2.72E-250
	orf02529	dacF	P38422丨DACF_BACSU	D-alanyl-D-alanine carboxypeptidase DacF	332/389(85.35)	4.56E-239
	orf00271	ansZ/yccC	O34482丨ASPG2_BACSU	L-asparaginase 2	278/377(73.74)	1.52E-193
	orf01170	aprE/apr/aprA/sprE	P00782丨SUBT_BACAM	Subtilisin E, extracellular alkaline serine protease	373/381(97.90)	1.49E-260
	orf04122	epr	P16396丨SUBE_BACSU	Minor extracellular protease Epr	326/579(56.30)	6.93E-206
	orf01634	npr	P06832丨NPRE_BACAM	Neutral metalloproteinase,Extracellular zinc metalloprotease	508/520(97.69)	0
	orf01694	bpr/bpf	P16397丨SUBF_BACSU	Bacillopeptidase F, extracellular serine-type endopeptidase activity	1004/1432(70.11)	0
	orf02032	ggt	P54422丨GGT_BACSU	Glutathione hydrolase proenzyme	492/585(84.10)	0
	orf02269	blaSE	P80057丨GSEP_BACLD	trypsin-like serine protease	76/227(33.48)	3.64E-24
	orf04089	vpr	P29141丨SUBV_BACSU	Minor extracellular protease Vpr,Serine protease	684/806(84.86)	0
Ribonuclease	orf01055	yhcR	P54602丨YHCR_BACSU	Endonuclease YhcR	439/645(68.06)	6.72E-288
	orf02388	yokF	O32001丨YOKF_BACSU	SPbeta prophage-derived endonuclease YokF	215/329(65.35)	2.35E-112
	orf03447	bsn	O32150丨BSN2_BACSU	Extracellular ribonuclease Bsn	270/288(93.75)	2.49E-207
	orf03743	unknown	P00648丨RNBR_BACAM	Ribonuclease	142/149(95.30)	1.10E-100
	orf02387	yobL	O34330丨YOBL_BACSU	HNH endonuclease	244/399(61.15)	2.40E-156
	orf01887	nucB	P42983丨NUCB_BACSU	Sporulation-specific extracellular nuclease	97/139(69.78)	2.16E-67
Pectate lyase	orf00828	pel	P39116丨PLY_BACSU	pectate lyase [Bacillus siamensis]	324/421(76.96)	7.06E-237
	orf03032	abnA	P94522丨EABN1_BACSU	Extracellular endo-alpha-(1->5)-L-arabinanase 1	266/320(83.12)	1.40E-205
	orf04191	pelB	P94449丨PEL2_BACIU	pectate lyase [Bacillus siamensis]	181/310(58.39)	7.51E-125
Xylanase	orf03941	xynA	P18429丨XYNA_BACSU	Endo-1,4-beta-xylanase A	202/213(94.84)	2.29E-152
	orf01992	xynD	Q45071丨XYND_BACSU	Arabinoxylan arabinofuranohydrolase	473/513(92.20)	0
	orf01991	xynC/ynfF	Q6YK37丨XYNC_BACIU	Glucuronoxylanase XynC	397/423(93.85)	1.7E-305

**Glucanase**	orf03034	ysdC	P94521丨YSDC_BACSU	Putative endo-1,4-beta-glucanase	326/361(90.30)	5.64E-243
	orf02122	ND	Q8J0I9丨GUN16_TRIHA	Endo-1,6-beta-D-glucanase BGN16.3	128/426(30.05)	1.39E-40
	orf04179	bglA	P07980丨GUB_BACAM	Beta-glucanase	223/239(93.31)	1.62E-175
**Lipase**	orf00225	glpQ	P37965丨GLPQ_BACSU	Glycerophosphodiester phosphodiesterase	237/293(80.89)	4.65E-174
	orf00273	estA	P37957丨ESTA_BACSU	triacylglycerol Lipase EstA	166/214(77.57)	1.68E-113
**Levanase**	orf04310	sacB	P21130丨SACB_BACAM	Levansucrase	456/472(96.61)	0
	orf04311	levB	O07003丨LEVB_BACSU	Levanbiose-producing levanase	393/512(76.76)	3.13e-311
**Amylase**	orf00301	amyE	P00691丨AMY_BACSU	Alpha-amylase	567/659(86.04)	0
**Chitosanase**	orf03473	csn	O07921丨CHIS_BACSU	Chitosanase	244/278(87.77)	5.86E-178
**Lactamase**	orf01334	penP	Q44674丨BLAC_BACAM	class A beta-lactamase [Bacillus siamensis]	270/304(88.82)	1.75E-195
**Cellulase**	orf01990	bglC/eglS	P07983丨GUN1_BACIU	Endoglucanase (cellulase)	474/499(94.99)	0
**Phytase**	orf02155	phy	O66037丨PHYT_BACSD	3-phytase	367/383(95.82)	3.22E-276
**Phosphatase**	orf01077	phoA	P19406丨PPB4_BACSU	Alkaline phosphatase 4	342/459(74.51)	2.09E-244

Table S7 Genes for putative plant growth promotion traits in the *Bacillus siamensis* YB-1631 genome

Function	Gene in strain YB-1631	Gene name	UniProt Accession Number	Description	Identity	E value
Indole-3-Acetic acid synthesis	*orf02090*	*dhaS*	O34660	aldehyde dehydrogenase	452/495(91.31)	0
	orf03290	*patB*	Q08432	L-Lysine--8-amino-7-oxononanoate transaminase	298/386(77.20)	4.08E-224
	orf01061	*yhcX*	P54608	N-acetyltransferase	488/513(95.13)	0
	orf00354	*bsdC*	P94405	Phenolic acid decarboxylase	454/473(95.98)	0
Cytokinin biosynthesis	*orf01899*	*miaA*	A7Z500	tRNA dimethylallyltransferase	300/314(95.54)	8.64E-212
	orf01868	*miaB*	A7Z4X2	tRNA-2-methylthio-N(6)-dimethylallyladenosine synthase	507/509(99.61)	0
Putrescine biosynthesis	*orf01627*	*speA*	P21885	Arginine decarboxylase	445/490(90.82)	0
	orf04025	*speB*	P70999	Agmatinase	277/290(95.52)	1.51E-209
Spermidine biosynthesis	*orf03052*	*speD(speH)*	A7Z7I7	S-adenosylmethionine decarboxylase	126/126(100.00)	2.22E-90
	orf04026	*speE*	P70998	Polyamine aminopropyltransferase	260/276(94.20)	3.8E-196
Acetoin, Butanediol biosynthesis	*orf03875*	*alsD*	Q04777	Alpha-acetolactate decarboxylase	209/255(81.96)	6.06E-162
	orf03876	*alsS*	Q04789	Acetolactate synthase	475/570(83.33)	0
	orf02974	*ilvH*	P37252	Acetolactate synthase small subunit	166/172(96.51)	5.58E-108
	orf02975	*ilvB*	P37251	Acetolactate synthase large subunit	513/574(89.37)	0
	orf00715	*bdhA*	O34788	(R,R)-butanediol dehydrogenase	324/346(93.64)	4.43E-237
Phosphate assimilation	*orf01077*	*phoA*	P19406	Alkaline phosphatase 4	342/459(74.51)	2.09E-244
	orf03062	*phoR*	P23545	Alkaline phosphatase synthesis sensor protein PhoR	411/577(71.23)	1.16E-287
	orf03755	*phoR*	P23545	Alkaline phosphatase synthesis sensor protein PhoR	85/229(37.12)	1.11E-42
	orf03063	*phoP*	P13792	Alkaline phosphatase synthesis transcriptional regulatory protein PhoP	210/240(87.50)	1.49E-148
	orf00264	*phoD*	P42251	Phosphodiesterase/alkaline phosphatase D	493/582(84.71)	0
Phosphate transport	*orf02706*	*pstS*	P46338	Phosphate-binding protein	252/300(84.00)	6.28E-175
	orf02705	*yqgH*	P46339	Phosphate transport system permease protein	274/309(88.67)	1.57E-184
	orf02702	*pstB1*	P46342	Phosphate import ATP-binding protein PstB 1	195/259(75.29)	5.62E-142
	orf02703	*pstB2*	P46341	Phosphate import ATP-binding protein PstB 2	226/269(84.01)	3.12E-157
	orf02704	*pstA*	P46340	Phosphate transport system permease protein	262/294(89.12)	1.94E-182
Nitrate/nitrite assimilation	*orf00326*	*nasD*	P42435	Nitrite reductase	764/805(94.91)	0
	orf00325	*nasE*	P42436	Assimilatory nitrite reductase [NAD(P)H] small subunit	94/106(88.68)	1.83E-70
	orf04017	*nasA*	P42432	Nitrate transporter	371/395(93.92)	1.03E-262
	orf00324	*nasF*	P42437	Uroporphyrinogen-III C-methyltransferase	346/472(73.31)	3.97E-262
Potassium assimilation	*orf03244*	*ktrA*	O32080	Ktr system potassium uptake protein A	190/222(85.59)	8E-137
	orf01612	*ktrC*	P39760	Ktr system potassium uptake protein C	209/221(94.57)	2.42E-142
	orf01495	*ktrD*	O31658	Ktr system potassium uptake protein D	394/450(87.56)	1.31E-272
Iron availability	*orf01361*	*yjkA*	O34684	Iron export ABC transporter permease subunit	210/248(84.68)	3.35E-132
	orf02534	*fur*	P54574	Ferric iron uptake transcriptional regulator	146/149(97.99)	1.06E-103
	orf03549	*yusV*	O32188	Probable siderophore transport system ATP-binding protein YusV	247/271(91.14)	1.33E-177
	orf03574	*yvrA*	O34631	ATP-binding cassette domain-containing protein	208/440(47.27)	1.65E-128
	orf03575	*yvrB*	O34451	ABC transporter permease	273/353(77.34)	2.66E-178
	orf03576	*yvrC*	O34805	ABC transporter substrate-binding protein	231/316(73.10)	2.34E-156

orf03586	*fhuC*	P49938	Iron(3+)-hydroxamate import ATP-binding protein FhuC	225/267(84.27)	2.69E-160
orf03587	*fhuG*	P49937	Iron ABC transporter permease	273/336(81.25)	9.88E-190
orf03588	*fhuB*	P49936	Iron(3+)-hydroxamate import system permease protein	267/339(78.76)	5.37E-187
orf03590	*fhuD*	P37580	Iron-hydroxamate ABC transporter substrate-binding protein	255/315(80.95)	5.44E-187
orf04111	*efeM*	P39596	Probable iron uptake system component EfeM	282/381(74.02)	6.32E-189
orf04112	*efeU*	P39595	Ferrous iron permease EfeU	367/482(76.14)	2.62E-244
orf04141	*fhuB*	P49936	iron ABC transporter permease	154/310(49.68)	6.07E-94
orf04142	*fhuG*	P49937	iron ABC transporter permease	141/335(42.09)	1.74E-88
orf04143	*feuA*	P40409	Iron-uptake system-binding protein	81/294(27.55)	7.33E-28
orf04211	*yxeB*	P54941	iron-hydroxamate ABC transporter substrate-binding protein	230/321(71.65)	1.89E-170
orf00174	*feuC*	P40411	Iron-uptake system permease protein FeuC	276/336(82.14)	1.27E-193
orf00175	*feuB*	P40410	Iron-uptake system permease protein FeuB	283/334(84.73)	1.44E-195
orf00176	*feuA*	P40409	Iron-uptake system-binding protein	260/318(81.76)	5.77E-182
orf00382	*yclN*	P94418	Petrobactin import system permease protein YclN	282/315(89.52)	1.73E-194
orf00383	*yclO*	P94419	iron chelate uptake ABC transporter family permease subunit	268/315(85.08)	2.03E-183
orf00384	*yclP*	P94420	Petrobactin import ATP-binding protein YclP	220/252(87.30)	5.38E-159
orf00312	*asbF*	Q81RQ4	Sugar phosphate isomerase/epimerase	72/270(26.67)	1.25E-22
orf00385	*yclQ*	P94421	ABC-type enterochelin transport system, periplasmic component	232/317(73.19)	6.43E-164
orf01120	*rhbE*	Q9Z3Q8	Lysine/ornithine N-monooxygenase	159/438(36.30)	5.1E-94
orf03228	*menF*	P23973	Isochorismate synthase EntC	335/470(71.28)	1.25E-246
orf03341	*ybdZ*	C0H3Q6	MbtH family protein, regulates adenylation domains of NRPSs	38/67(56.72)	2.8E-24
orf03342	*dhbF*	P45745	Acyl carrier protein丨EntF, seryl-AMP synthase component of non-ribosomal peptide synthetase丨Thioesterase domain of type I polyketide synthase or non-ribosomal peptide synthetase	1657/2378(69.68)	0
orf03343	*dhbB*	P45743	Isochorismate hydrolase丨Acyl carrier protein/domain	227/314(72.29)	3.84E-167
orf03344	*dhbE*	P40871	EntE, 2,3-dihydroxybenzoate-AMP synthase component of non-ribosomal peptide synthetase	435/533(81.61)	0
orf03345	*dhbC*	P45744	Isochorismate synthase EntC	260/395(65.82)	1.03E-187
orf03346	*dhbA*	P39071	NAD(P)-dependent dehydrogenase, short-chain alcohol dehydrogenase family	186/261(71.26)	4.31E-128
orf03549	*yusV*	O32188	ABC-type cobalamin/Fe3+-siderophores transport system, ATPase component	247/271(91.14)	1.33E-177
orf03574	*yvrA*	O34631	ABC-type cobalamin/Fe3+-siderophores transport system, ATPase component丨Adenosylcobinamide amidohydrolase	208/440(47.27)	1.65E-128
orf03575	*yvrB*	O34451	ABC-type Fe3+-siderophore transport system, permease component	273/353(77.34)	2.66E-178
orf03586	*fhuC*	P49938	ABC-type cobalamin/Fe3+-siderophores transport system, ATPase component	225/267(84.27)	2.69E-160

orf03587	*fhuG*	P49937	ABC-type Fe3+-siderophore transport system, permease component	273/336(81.25)	9.88E-190
orf03588	*fhuB*	P49936	ABC-type Fe3+-siderophore transport system, permease component	267/339(78.76)	5.37E-187
orf04141	*fhuB*	P49936	ABC-type Fe3+-siderophore transport system, permease component	154/310(49.68)	6.07E-94
orf04142	*fhuG*	P49937	ABC-type Fe3+-siderophore transport system, permease component	141/335(42.09)	1.74E-88
orf00385	*yclQ*	P94421	siderophore ABC transporter substrate-binding protein	232/317(73.19)	6.43E-164

Table S8 Genes for putative plant-microbe interaction in the *Bacillus siamensis* YB-1631 genome

Function	Gene in strain YB-1631	Gene name	UniProt Accession Number	Description	Identity	E value
Bacterial chemotaxis	*orf01811*	*cheA*	P29072	Chemotaxis protein CheA	581/677(85.82)	0
	orf01812	*cheW*	P39802	Chemotaxis protein CheW	125/151(82.78)	2.13E-84
	orf01813	*cheC*	P40403	CheY-P phosphatase CheC	186/209(89.00)	4.34E-130
	orf01814	*cheD*	A7Z4R7	Chemoreceptor glutamine deamidase CheD	164/166(98.80)	4.01E-114
	orf02452	*cheR*	P31105	Chemotaxis protein methyltransferase	208/256(81.25)	3.08E-151
	orf01801	*cheY*	P24072	Chemotaxis protein CheY	113/120(94.17)	1.93E-74
	orf01561	*CheV*	P37599	Chemotaxis protein CheV	256/303(84.49)	1.16E-183
	orf03260	*tlpB*	P39217	Methyl-accepting chemotaxis protein TlpB	373/552(67.57)	3.72E-217
	orf03261	*mcpA*	P39214	Methyl-accepting chemotaxis protein McpA	428/586(73.04)	1.74E-290
	orf03262	*mcpA*	P39214	Methyl-accepting chemotaxis protein McpA	335/590(56.78)	2.22E-219
	orf03263	*mcpB*	P39215	Methyl-accepting chemotaxis protein McpB	410/551(74.41)	7.36E-222
Flagellar assembly/Motility	*orf01785*	flgB	P24500	Flagellar basal body rod protein FlgB	95/129(73.64)	4.79E-61
	orf01786	flgC	P24501	Flagellar basal-body rod protein FlgC	129/150(86.00)	1.12E-85
	orf01787	fliE	P24502	Flagellar hook-basal body complex protein FliE	93/106(87.74)	3.92E-47
	orf01788	fliF	P23447	Flagellar M-ring protein	418/536(77.99)	9E-275
	orf01789	fliG	P23448	Flagellar motor switch protein FliG	320/338(94.67)	2.39E-221
	orf01790	fliH	P23449	Probable flagellar assembly protein FliH	151/206(73.30)	8.35E-99
	orf01791	fliI	P23445	Flagellum-specific ATP synthase	403/438(92.01)	3.02E-286
	orf01792	fliJ	P20487	Flagellar FliJ protein	132/147(89.80)	3.51E-83
	orf01793	ylxF	P23454	FlaA locus 22.9 kDa protein	142/203(69.95)	1.66E-44
	orf01794	fliK	P23451	Probable flagellar hook-length control protein	227/460(49.35)	8.46E-118
	orf01795	ylxG	P23455	FlaA locus uncharacterized protein YlxG	92/144(63.89)	3.76E-57
	orf01796	flgG	P23446	Flagellar basal-body rod protein FlgG	189/264(71.59)	4.05E-126
	orf03918	flhP	P39753	Flagellar hook-basal body complex protein FlhP	187/273(68.50)	1.71E-123
	orf03919	flhO	P39752	Flagellar hook-basal body protein	219/270(81.11)	1.19E-142
	orf01798	fliL	P23452	Flagellar protein FliL	102/140(72.86)	1.12E-57
	orf01799	fliM	P23453	Flagellar motor switch protein FliM	312/330(94.55)	1.44E-219
	orf01800	fliY	P24073	Flagellar motor switch phosphatase FliY	316/382(82.72)	1.11E-200
	orf01802	fliZ	P35536	Flagellar biosynthetic protein FliZ	158/210(75.24)	7.26E-94
	orf01803	fliP	P35528	Flagellar biosynthetic protein FliP	205/221(92.76)	1.67E-133
	orf01804	fliQ	P35535	Flagellar biosynthetic protein FliQ	74/81(91.36)	1.11E-43
	orf01805	fliR	P35537	Flagellar biosynthetic protein FliR	222/259(85.71)	6E-148
	orf01806	flhB	P35538	Flagellar biosynthetic protein FlhB	311/359(86.63)	1.97E-217
	orf01807	flhA	P35620	Flagellar biosynthesis protein FlhA	618/677(91.29)	0
	orf01808	flhF	Q01960	Flagellar biosynthesis protein FlhF	236/365(64.66)	4.64E-160
	orf03806	hag	P02968	Flagellin Hag	187/305(61.31)	2.24E-96
	orf01809	ylxH	P40742	Flagellum site-determining protein YlxH	198/291(68.04)	2.03E-137
	orf01514	motB	P28612	Flagellar motor protein MotB	216/255(84.71)	1.82E-143
	orf01515	motA	P28611	Flagellar motor component MotA	245/271(90.41)	5.66E-161

	orf01816	swrB	P40405	Swarming motility protein SwrB	94/167(56.29)	1.13E-47
	orf01797	swrD	C0H412	Swarming motility protein SwrD	60/71(84.51)	9.5E-39
	orf03791	SwrAA	O32266	Swarming motility protein SwrAA	99/111(89.19)	6.22E-68
	orf00773	swrC	O31501	Swarming motility protein SwrC	907/1052(86.22)	0
Biofilm formation	orf04310	sacB	P21130丨SACB_BACAM	Levan sucrase	456/472(96.61)	0
	orf00284	znuA	O34966丨ZNUA_BACSU	High affinity zinc ABC transporter lipoprotein	255/319(79.94)	5.69E-180
	orf00809	yfnF	O06484丨YFNF_BACSU	Collagen like triple helix with GXT repeats5	234/303(77.23)	1.26E-187
	orf00806	yfnI	Q797B3丨LTAS1_BACSU	Collagen like triple helix with GXT repeats4	546/636(85.85)	0
	orf04104	slrA	P0C8M5	DNA-binding anti-repressor SinI	30/52(57.69)	1.99E-12
	orf04103	ywcC	P39601	TetR/AcrR family transcriptional regulator	126/200(63.00)	5.06E-90
	orf00005	remB	P37525	Extracellular matrix regulatory protein B	70/81(86.42)	2.61E-45
	orf03833	lytC	Q02114	N-acetylmuramoyl-L-alanine amidase	297/492(60.37)	2.45E-204
	orf03834	lytB	Q02113	Amidase enhancer	398/692(57.51)	1.24E-271
	orf03835	lytA	Q02112	Membrane-bound protein LytA	47/105(44.76)	3.94E-20
	orf00043	abrB	P08874	Transition state regulatory protein AbrB	93/94(98.94)	1.85E-58
	orf01572	ykzF	O31697	Antirepressor AbbA	58/63(92.06)	1.31E-35
	orf00038	yaaT	P37541	Cell fate regulator YaaT, PSP1 superfamily (controls sporulation, competence, biofilm development)	265/275(96.36)	2.73E-184
	orf00908	yfiQ	O31559	Membrane-bound acyltransferase YfiQ, involved in biofilm formation	236/352(67.05)	6.37E-159
	orf01049	yhcK	P54595	GGDEF domain, diguanylate cyclase (c-di-GMP synthetase) or its enzymatically inactive variants	218/359(60.72)	2.2E-148
	orf01116	RBAM_010030	A7Z2Z1	Cell fate regulator YlbF, YheA/YmcA/DUF963 family (controls sporulation, competence, biofilm development)	114/116(98.28)	5.63E-72
	orf01664	ylbF	O34412	Cell fate regulator YlbF, YheA/YmcA/DUF963 family (controls sporulation, competence, biofilm development)	132/149(88.59)	5.87E-92
	orf01734	remA	Q7WY72	Extracellular matrix regulatory protein A	89/89(100.00)	9.61E-56
	orf01815	sigD	P10726	Regulatory protein YlbF	247/254(97.24)	5.96E-173
	orf01869	ymcA	O31779	Cell fate regulator YmcA, YheA/YmcA/DUF963 family (controls sporulation, competence, biofilm development)	133/143(93.01)	1.3E-83
	orf02401	ypqE	P50829	Phosphotransferase system IIA component	124/168(73.81)	7.68E-88
	orf02667	sinI	P23308	DNA-binding anti-repressor SinI	39/57(68.42)	2.08E-18
	orf02668	sinR	P06533	unnamed protein product	110/111(99.10)	6.02E-72
	orf02669	tasA	P54507	Spore coat protein	219/261(83.91)	1.09E-151
	orf02670	sipW	P54506	Signal peptidase I	143/189(75.66)	1.37E-69

	orf02671	tapA	P40949	TasA anchoring/assembly protein	95/176(53.98)	3.02E-71
	orf03011	yshB	P94543	Colicin V production accessory protein CvpA, regulator of purF expression and biofilm formation	143/177(80.79)	9.44E-91
	orf03111	ytrP	O34325	GGDEF domain, diguanylate cyclase (c-di-GMP synthetase) or its enzymatically inactive variants	371/578(64.19)	1.59E-257
	orf03214	luxS	A7Z800	S-ribosylhomocysteine lyase LuxS, autoinducer biosynthesis	157/157(100.00)	1.73E-115
	orf03242	bslA	P71014	Biofilm-surface layer protein A	134/180(74.44)	4.33E-86
	orf03319	degQ	P06532	Degradation enzyme regulation protein DegQ	45/46(97.83)	6.52E-24
	orf03690	sigL	P24219	DNA-directed RNA polymerase specialized sigma subunit, sigma54 homolog	317/435(72.87)	7.95E-223
	orf03692	epsO	P71065	Putative pyruvyl transferase EpsO	218/320(68.12)	2.89E-163
	orf03693	epsN	Q795J3	UDP-N-acetylbacillosamine N-acetyltransferase	288/386(74.61)	4.19E-206
	orf03694	epsM	P71063	acetyltransferase	59/112(52.68)	6.49E-34
	orf03695	epsL	P71062	sugar transferase	154/199(77.39)	2.61E-107
	orf03696	epsK	P71060	MATE family efflux transporter	389/505(77.03)	7.8E-272
	orf03697	epsJ	P71059	glycosyltransferase	221/343(64.43)	1.03E-160
	orf03698	epsI	P71058	polysaccharide pyruvyl transferase family protein	272/358(75.98)	3.3E-199
	orf03699	epsH	P71057	glycosyltransferase family 2 protein	241/345(69.86)	2.31E-176
	orf03700	epsG	P71056	EpsG family protein	313/367(85.29)	1.45E-232
	orf03701	epsF	P71055	glycosyl transferase	254/381(66.67)	5.24E-184
	orf03702	epsE	P71054	Glycosyltransferase family 2 protein	236/280(84.29)	2.99E-174
	orf03703	epsD	P71053	Glycosyltransferase family 4 protein	278/381(72.97)	3.32E-203
	orf03704	epsC	P71052	Polysaccharide biosynthesis protein	490/597(82.08)	0
	orf03705	yveL	P71051	CpsD/CapB family tyrosine-protein kinase	198/225(88.00)	4.18E-132
	orf03706	yveK	P71050	hypothetical protein	156/235(66.38)	5.47E-104
	orf03707	slrR	P71049	Helix-turn-helix domain-containing protein	119/150(79.33)	6.35E-79
	orf03808	csrA	A7Z9A6	sRNA-binding carbon storage regulator CsrA	74/74(100.00)	6.48E-45
	orf03837	mna A	P39131	UDP-N-acetylglucosamine 2-epimerase	309/378(81.75)	2.09E-229
	orf03844	tagA	P27620	UDP-N-acetyl-D-mannosaminuronic acid transferase, WecB/TagA/CpsF family	169/256(66.02)	4.02E-126
γ-Polyglutamic acid synthesis	*orf03863*	capB, pgsB, ywsC	P96736	poly-gamma-glutamate synthase PgsB	1176/1182(99.5)	0
	orf03862	capC, pgsC	P96737	PGA biosynthesis protein CapC	447/450(99.3)	0
	orf03861	capA, pgsA	P96738	Poly-gamma-glutamate biosynthesis protein CapA	298/380(78.42)	2.69E-223
	orf03860	pgsE, ywtc	P96739	Uncharacterized protein, required for polyglutamate synthesis	36/48(75.00)	2.04E-19
	orf02989	murI	A7Z7C8	glutamate racemase	259/271(95.57)	4.76E-190

Bacterial target molecules for general plant immune response	orf03812	flgK	P39810丨FLGK_BACSU	Flagellin HAP1	315/507(62.13)	1.60E-212
	orf03805	fliD	P39738丨FLID_BACSU	Flagellin HAP2	255/513(49.71)	4.44E-147
	orf03806	hag	P02968丨FLA_BACSU	Flagellin HAG	187/305(61.31)	2.24E-96
	orf00124	tufA	A7Z0N5丨EFTU_BACVZ	Elongation factor EF-Tu	395/396(99.75)	2.31E-287
Lipopolysaccharide biosynthesis (elicitation of plant basal defence)	orf03751	trxB	P80880丨TRXB_BACSU	thioredoxin-disulfide reductase	298/315(94.60)	4.17E-218
	orf03752	cwlO	P40767丨CWLO_BACSU	NlpC/P60 family protein	382/473(80.76)	3.86E-146
	orf03754	yvcD	O06968丨YVCD_BACSU	tetratricopeptide repeat protein	409/484(84.50)	2.77E-303
	orf03755	RBAM_032040	P23545丨PHOR_BACSU	HAMP domain-containing histidine kinase	85/229(37.12)	1.11E-42
	orf03756	RBAM_032050	Q49ZT8丨HSSR_STAS1	response regulator transcription factor	106/220(48.18)	2.42E-66
	orf03759	bmrA	O06967丨BMRA_BACSU	ABC transporter ATP-binding protein	504/588(85.71)	0
	orf03760	hisIE	O34912丨HIS2_BACSU	bifunctional phosphoribosyl-AMP cyclohydrolase/phosphoribosyl-ATP diphosphatase HisIE	180/208(86.54)	8.68E-127
	orf03761	hisF	A7Z962丨HIS6_BACVZ	imidazole glycerol phosphate synthase subunit HisF	245/252(97.22)	1.53E-174
	orf03762	hisA	A7Z963丨HIS4_BACVZ	1-(5-phosphoribosyl)-5-((5-phosphoribosylamino)methylideneamino)imidazole-4-carboxamide isomerase	233/245(95.10)	8.08E-162
Teichuronic acid/lipopoly-saccharide biosynthesis (elicitation of plant basal defence)	orf03832	tuaA	O32274丨TUAA_BACSU	exopolysaccharide biosynthesis polyprenyl glycosylphosphotransferase	121/160(75.62)	2.29E-79
	orf03831	tuaB	O32273丨TUAB_BACSU	MOP flippase family protein	391/482(81.12)	1.35E-258
	orf03830	tuaC	O32272丨TUAC_BACSU	glycosyltransferase family 4 protein	251/384(65.36)	7.78E-183
	orf03829	tuaD	O32271丨TUAD_BACSU	UDP-glucose/GDP-mannose dehydrogenase family protein	329/434(75.81)	1.62E-240
	orf03828	tuaE	O32270丨TUAE_BACSU	O-antigen ligase family protein	321/481(66.74)	3.62E-216
	orf03827	tuaF	O32269丨TUAF_BACSU	Teichuronic acid biosynthesis protein TuaF	154/226(68.14)	5.09E-105
	orf03826	tuaG	O32268丨TUAG_BACSU	glycosyltransferase family 2 protein	214/252(84.92)	4.82E-162
	orf03825	tuaH	O32267丨TUAH_	glycosyltransferase	260/393(66.16)	1.59E-198

orf03824	tagO	BACSU O34753|TAGO_ BACSU	utative undecaprenyl-phosphate N-acetylglucosaminyl 1-phosphate transferase	278/306(90.85)	2.47E-185

Table S9 Genes for breakdown, transport and utilization of plant derived substrates in the *Bacillus siamensis* YB-1631 genome

Function	Gene in strain YB-1631	Gene name	UniProt Accession Number	Description	Identity	E value
Cellulose and hemicellulose	orf03941	xynA	P18429丨XYNA_BACSU	xylanase	202/213(94.84)	2.29E-152
	orf01992	xynD	Q45071丨XYND_BACSU	xylanase	473/513(92.20)	0
	orf04158	xynP	P94360丨MSMX_BACSU	hypothetical symporter	335/365(91.78)	3.79E-241
	orf01918	xynB	P94489丨XYNB_BACSU	Xylan beta-1,4-xylosidase	504/533(94.56)	0
	orf01346	lacR	P18816丨LACR_LACLL	lactose phosphotransferase system repressor protein	112/251(44.62)	4.27E-66
	orf01345	lacG	C7N8L9丨LACG_LEPBD	Putative 6-phospho-beta-galactosidase	284/464(61.21)	3.15E-221
	orf01343	lacE	P50976丨PTLCB_STRMU	Phosphotransferase system	314/567(55.38)	9.82E-216
	orf01344	lacF	P26426丨PTLA_STRMU	Phosphotransferase system	54/101(53.47)	1.42E-32
	orf04101	galK1	P39574丨GAL1_BACSU	Galactokinase galK1	274/390(70.26)	1.93E-201
	orf04164	galE1	P55180丨GALE_BACSU	UDP-glucose 4-epimerase	306/338(90.53)	1.26E-230
	orf04100	galT1	P39575丨GALT_BACSU	Galactose-1-phosphate uridyltransferas	358/511(70.06)	8.69E-280
Chitin, chitosan	orf03473	csn	O07921丨CHIS_BACSU	chitosanase	244/278(87.77)	5.86E-178
Protein, peptide	orf01694	bpr	P16397丨SUBF_BACSU	Bacillopeptidase F	1004/1432(70.11)	0
	orf02032	ggt	P54422丨GGT_BACSU	glutamyltranspeptidase	492/585(84.10)	0
Opine	orf00350	tcyB	P42200丨TCYB_BACSU	L-cystine transport system permease protein TcyB	209/229(91.27)	2.26E-143
	orf00351	yckK	P42199丨TCYA_BACSU	Probable amino-acid ABC transporter extracellular binding protein	225/268(83.96)	1.88E-157
	orf00366	yckJ	P54952丨YXEM_BACSU	HisM, ABC-type amino acid transport system, permease	233/264(88.26)	5.73E-162
	orf00367	yckI	P54953丨YXEN_BACSU	ABC-type polar amino acid transport system	201/224(89.73)	7.98E-138
	orf00368	ytmJ	P54954丨YXEO_BACSU	amino acid ABC transporter, amino acid-binding protein	212/249(85.14)	9.83E-149
	orf03633	ytmK	O34852丨TCYK_BACSU	amino acid ABC transporter, amino acid-binding protein, His/Glu/Gln/Arg/opine family	211/270(78.15)	7.14E-150
	orf03632	ytmL	O34315丨TCYL_BACSU	putative amino-acid ABC transporter ATP-binding protein	191/239(79.92)	7.15E-128
	orf03631	ytmM	O34931丨TCYM_BACSU	Amino acid ABC transporter, permease protein, 3-TM	176/233(75.54)	2.09E-120
	orf03630	ytmN	O34900丨TCYN_BACSU	HisP and GlnQ are the ATP-binding components of the bacterial periplasmic histidine and glutamine permeases	214/259(82.63)	2.80E-153
	orf00366	yxeN	P54952丨YXEM_BACSU	Amino acid ABC transporter, permease protein, 3-TM region, His/Glu/Gln/Arg/opine	233/264(88.26)	5.73E-162

	orf00367	RBAM03043	P54953丨YXEN_BACSU	COG2226, UbiE, Methylase involved in ubiquinone/menaquinone biosynthesis	201/224(89.73)	7.98E-138
	orf00368	yurR	P54954丨YXEO_BACSU	COG0665, DadA, Glycine/D-amino acid oxidases (deaminating)	212/249(85.14)	9.83E-149
	orf03583	RBAM03038	C0H3R5丨YVRJ_BACSU	Helix-turn-helix transcription regulator	44/50(88.00)	3.71E-22
	orf00193	yurP	E0U070丨GLMS_BACPZ	AgaS, Predicted phosphosugar isomerase	559/600(93.17)	0
	orf03462	yurO	O32156丨YURO_BACSU	ABC-type sugar transport system, periplasmic component	401/421(95.25)	1.54E-296
	orf03461	yurN	O32155丨YURN_BACSU	ABC sugar transporter, inner membrane subunit	283/291(97.25)	3.74E-195
	orf03460	yurM	O32154丨YURM_BACSU	ABC sugar transporter, inner membrane subunit	294/300(98.00)	5.05E-213
	orf03459	yurL	O32153丨FRLD_BACSU	cd01940, Fructose-lysine_kinase_like	262/285(91.93)	9.99E-194
	orf03458	yurK	O32152丨YURK_BACSU	pfam00392, GntR, Bacterial regulatory proteins	231/242(95.45)	2.18E-169
	orf03457	yurJ	O32151丨YURJ_BACSU	MalK, ABC-type sugar transport systems, ATPase component	342/367(93.19)	7.20E-249

Table S10. Genes for putative response to environmental stress in the *Bacillus siamensis* YB-1631 genome

Function	Gene in strain YB-1631	Gene name	UniProt Accession Number	Description	Identity	E value
oxidative stress	orf03094	tpx	P80864丨TPX_BACSU	Thiol peroxydase	157/167(94.01)	5.90E-112
	orf02709	sodA	P54375丨SODM_BACSU	Superoxide dismutas	187/202(92.57)	1.44E-138
nucleic acid-binding protein	orf00524	cspC	P39158丨CSPC_BACSU	Cold shock protein	64/65(98.46)	6.19E-42
	orf01047	cspB	P32081丨CSPB_BACSU	Major cold shock protein	64/65(98.46)	2.12E-42
	orf02180	cspD	P51777丨CSPD_BACSU	Cold shock protein	65/66(98.48)	3.56E-43
nucleic acid-modification	orf01028	cspR	O31590丨TRML_BACSU	Putative rRNA methylase	144/156(92.31)	3.87E-106

Table S11. Comparison of genes for antimicrobial compounds in the genomes of five *B. siamensis* isolates

Type	Metabolites	*B. siamensis* KCTC 13613^T	*B. siamensis* SCSIO 05746	*B. siamensis* JFL15	*B. siamensis* RGM 2529	*B. siamensis* YB-1631
Lipopeptides	Surfactin	20	20	20	20	20
	Fengycin	14	15	15	15	14
	Bacillomycin D/iturin	10	10	10	10	10
	Bacillibactin	13	13	13	13	13
	Locillomycin	5	4	5	5	5
Polyketides	Bacillaene	14	13	14	14	14
	Macrolactin	0	10	0	0	0
	Difficidin	15	15	15	1	15
	Aurantinin	0	0	0	19	0
cyclic peptides	Amylocyclicin	6	6	6	6	6
	Uberolysin/carnocyclin family circular bacteriocin	1	1	1	1	1
Dipeptides	Bacilysin	1	7	1	1	1
Cationic peptide	Lci	1	1	1	1	1
Linear azol(in)e-containing peptides	Plantazolicin	0	0	11	0	0
Aminoglycoside	Butirosin	2	2	2	2	2

Table S12. Comparison of genes for extracellular hydrolases in the genomes of five *B. siamensis* isolates

Type	*B. siamensis* KCTC 13613^T	*B. siamensis* SCSIO 05746	*B. siamensis* JFL15	*B. siamensis* RGM 2529	*B. siamensis* YB-1631
Peptidase	4	4	4	4	4
Amidase	6	6	6	6	6
Glycosidases/glycosyltransferases	3	3	3	3	3
Protease	9	9	9	9	10
Ribonuclease	4	5	6	4	6
Pectate lyase	3	3	3	3	3
Xylanase	3	3	3	3	3
Glucanase	3	3	3	3	3
Lipase	2	2	2	2	2
Levanase	2	2	2	2	2
Amylase	1	1	1	1	1
Chitosanase	1	1	1	1	1
Lactamase	1	1	1	1	1
Cellulase	1	1	1	1	1

Table S13. Comparison of genes for putative plant growth promotion traits in the genomes of five *B. siamensis* isolates

Type	*B. siamensis* KCTC 13613^T	*B. siamensis* SCSIO 05746	*B. siamensis* JFL15	*B. siamensis* RGM 2529	*B. siamensis* YB-1631
Indole-3-Acetic acid synthesis	5	5	5	5	5
Cytokinin biosynthesis	3	3	3	3	3
Putrescine biosynthesis	2	2	2	2	2
Spermidine biosynthesis	2	2	2	2	2
Acetoin biosynthesis	2	2	2	2	2
Butanediol biosynthesis	3	3	3	3	3
Phosphate assimilation	12	12	12	12	12
Nitrate/nitrite assimilation	10	10	10	10	10
Potassium assimilation	3	3	3	3	3
Iron availability	31	31	31	29	31

Table S14. Comparison of genes for putative plant-microbe interaction and bacterial survival traits in the genomes of five *B. siamensis* isolates

Type	Function	*B. siamensis* KCTC 13613^T	*B. siamensis* SCSIO 05746	*B. siamensis* JFL15	*B. siamensis* RGM 2529	*B. siamensis* YB-1631
**Plant microbe interactions**	Chemotaxis	11	11	11	11	11
	Flagellar assembly/Motility	32	32	32	32	32
	Biofilm	56	56	57	52	56
	Involved in triggering plant immune response	22	22	22	22	22
**Bacterial survival traits**	Utilization of plant derived substrates	34	34	34	34	34
	Response to environmental stress	6	6	6	6	6
